# High Resolution Multiview Holographic Display Based on the Holographic Optical Element

**DOI:** 10.3390/mi14010147

**Published:** 2023-01-06

**Authors:** Xiujuan Qin, Xinzhu Sang, Hui Li, Rui Xiao, Chongli Zhong, Binbin Yan, Zhi Sun, Yu Dong

**Affiliations:** 1 State Key Laboratory of Information Photonics and Optical Communications, Beijing University of Posts and Telecommunications, Beijing 100876, China; 2 Beijing National Research Center for Information Science and Technology, Tsinghua University, Beijing 100084, China

**Keywords:** holographic display, spatial light modulator, holographic optical element

## Abstract

Limited by the low space-bandwidth product of the spatial light modulator (SLM), it is difficult to realize multiview holographic three-dimensional (3D) display. To conquer the problem, a method based on the holographic optical element (HOE), which is regarded as a controlled light element, is proposed in the study. The SLM is employed to upload the synthetic phase-only hologram generated by the angular spectrum diffraction theory. Digital grating is introduced in the generation process of the hologram to achieve the splicing of the reconstructions and adjust the position of the reconstructions. The HOE fabricated by the computer-generated hologram printing can redirect the reconstructed images of multiview into multiple viewing zones. Thus, the modulation function of the HOE should be well-designed to avoid crosstalk between perspectives. The experimental results show that the proposed system can achieve multiview holographic augmented reality (AR) 3D display without crosstalk. The resolution of each perspective is 4K, which is higher than that of the existing multiview 3D display system.

## 1. Introduction

Recently, AR has drawn significant attention due to widespread application in various fields, such as industry, medicine, and entertainment [1,2,3,4,5,6]. The ideal goal of AR display is to present a virtual image superimposed on a real-world scene and avoid visual fatigue [7]. Holographic display can reconstruct 3D images with real physical depth cues and high resolution [8,9,10,11], which makes it a strong candidate for AR display. However, the narrow field of view (FOV) caused by low product of the space-bandwidth of the SLM has always been a significant barrier that promotes the development of AR display [12,13].

The enhanced viewing angle can be achieved by multiview 3D display [14,15,16]. The method of realizing multiview 3D display can be classified into geometric optics method and diffractive optics method. Multiview 3D display based on geometric optics mainly contains a parallax barrier, lenticular lenses, and micro lenses. It can provide parallax information for observers, which has the advantage of low cost and a small amount of refresh data. However, there are a series of problems, such as large crosstalk, limited number of viewing zones, and limited resolution for each perspective [17,18,19].

In recent years, many methods to achieve multiview holographic display based on the diffractive optical element have been proposed. Linsen Chen [20] et al. proposed a multiview 3D display based on four-level pixelated blazed gratings, and four views with a resolution of 100 × 100 were observed. They also designed an optical combiner based on pixelated volume holographic optical element (P-VHOE) for multiview 3D display [21]. P-VHOE reconstructed four views with a resolution of 200 × 200. AR 3D display with high diffraction efficiency, high transmittance, and natural motion parallax was achieved. Yanfeng Su [22] et al. proposed multiview holographic 3D display using an SLM and a directional diffractive device. Crosstalk-free multiview holographic 3D display with four views was successfully achieved, in which the resolution of each view was 30 × 30. Meanwhile, they also proposed an optimized method of eight-view holographic 3D display by using two SLMs and a large-area directional diffractive device to enhance the resolution for each view. The resolution of each view was 60 × 60 [23]. However, in the methods proposed above, the resolution of each viewpoint is low.

Here, we propose a multiview holographic 3D display system with a phase SLM and a directional controlling element HOE. The phase SLM is employed to upload the synthetic phase hologram to achieve seamless splicing and no crosstalk of the reconstructed images. The main function of the core component HOE is redirecting the multiview reconstructed images into multiple viewing zones to realize multiview display. First, the HOE is designed based on the point source method according to the position of the four different focal points. Second, the complex amplitude distribution of the hologram with four different focal points is superimposed and divided into four different parts. Here, the resolution of each part is 1920 × 1080. Third, the hologram for HOE is uploaded onto the SLM, and the virtual hologram is generated through a 4*f* optical system. Then, the HOE is fabricated by computer-generated hologram printing technique. Finally, the probe waves with the information from four perspectives are projected onto the HOE to realize multiview display. The experimental results demonstrate that our proposed system can achieve multiview holographic AR 3D display without crosstalk. Meanwhile, the resolution of each perspective is 4K, which is higher than that of the existing multiview holographic display system. Furthermore, the width of the viewing zone for each perspective is 2 cm when the viewing distance is 25 cm.

## 2. Designed System and the Proposed Method

### 2.1. Schematic of the Multiview Holographic Display

**Figure 1:**
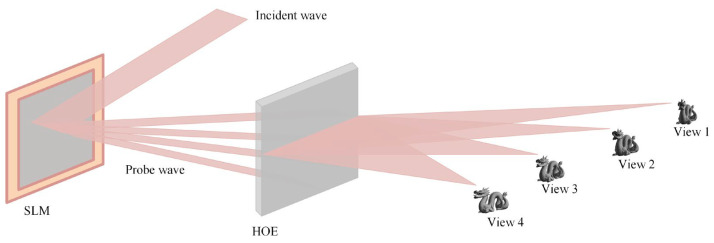
The schematic of the multiview holographic 3D display.

The schematic of the multiview holographic display is illustrated in Figure 1. The incident wave is modulated by the phase SLM, on which the 4-view images are generated with different directions. To design an optical combiner HOE with the function of multiview display, four lens functions with different focal points are superimposed, and each lens function is calculated by the point source method. In the process of calculating lens function, the direction of reference wave for each lens is different. The HOE is generated using computer-generated hologram printing. In the reconstruction process, the recorded HOE can modulate 4-view reconstructed images into specific positions, respectively, based on the angular selectivity. The detailed design parameters of the HOE are shown in Table 1.

### 2.2. Design Process of the HOE

**Figure 2:**
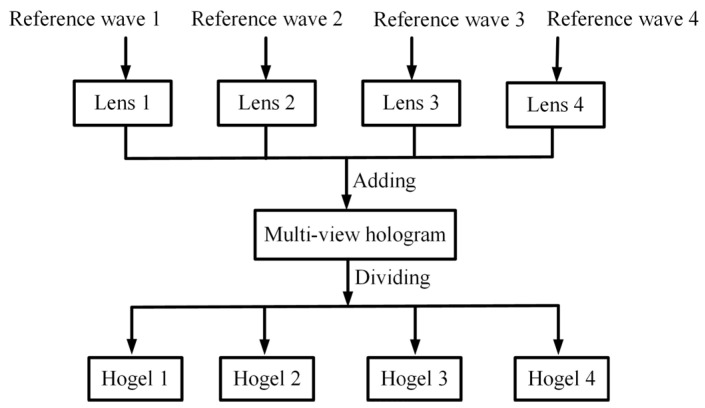
The designed algorithm of HOE.

The design algorithm of HOE is based on the point source method and is shown in Figure 2. Firstly, four lens functions with different focal points are calculated. The complex amplitude distribution of each lens $E_{i}(x,y)$ on the hologram plane can be described as follows:

(1) $$E_{i}(x,y)=\frac{\exp(j\cdot k\cdot \sqrt{{\left((x\cdot p)-a_{i}\right)}^{2}+{\left((y\cdot p)-b\right)}^{2}+z^{2}})}{\sqrt{{\left((x\cdot p)-a_{i}\right)}^{2}+{\left((y\cdot p)-b\right)}^{2}+z^{2}}}$$

where *k* is the wave vector and k=2π/λ, *λ* is the wavelength of the incident wave. *p* is the pixel pitch of the SLM. i(i=1,2,3,4) represents four different lenses. *z* represents the focal length of the lens. $\left(a_{i},b\right)$ denotes the focal points of lens. In this study, the coordinates of four different focal points should be set appropriately to avoid crosstalk and are illustrated in Section 2.1.

Secondly, four reference waves with different directions are introduced in the generation process of the hologram to satisfy angular selectivity. The complex amplitude distribution of each lens $E_{i}(x,y)$ is calculated by:

(2) $$E_{i}(x,y)=\frac{\exp(j\cdot k\cdot \sqrt{{\left((x\cdot p).\cos(\theta_{i})-a_{i}\right)}^{2}+{\left((y\cdot p)-b\right)}^{2}+z^{2}})}{\sqrt{{\left((x\cdot p).\cos(\theta_{i})-a_{i}\right)}^{2}+{\left((y\cdot p)-b\right)}^{2}+z^{2}}}$$

where $\theta_{i}$ denotes the direction of the reference wave. Thirdly, the complex amplitude distribution of the hologram with four different focal points is superimposed. To ensure the contents of the reconstructed image are not lost, the computer-generated hologram (CGH) of the HOE has the resolution of 3840×2160. Here, the hologram is divided into four sub-holograms (hogels) with resolution of 1920×1080, and each hogel should be sequentially recorded.

### 2.3. The Process of Manufacturing HOE Using Computer-Generated Hologram Printing Technique

**Figure 3:**
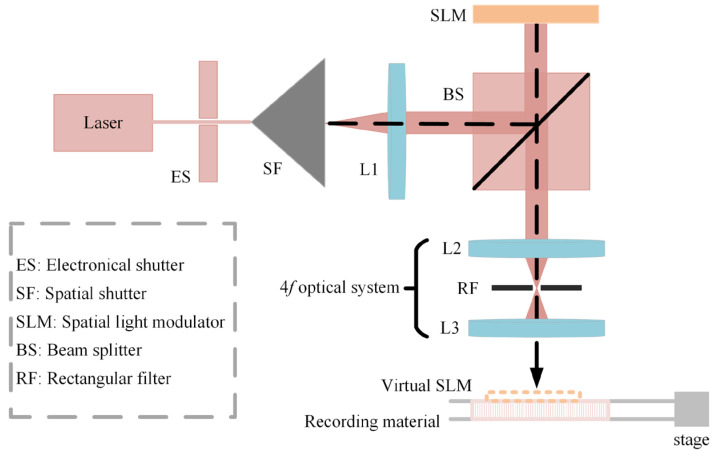
The setup for fabricating the HOE.

As presented in Section 2.2, the pixel number of the phase SLM (resolution with 1920×1080) is insufficient to display an entire CGH at once. Therefore, the CGH should be divided into a set of hogels. The experimental setup for generating optical combiner HOE is shown in Figure 3. ES is used to control the exposure time to obtain maximum diffraction efficiency. The reference wave illuminates the SLM via BS. Then, the wavefront information from the SLM is introduced into the 4*f* optical system. The rectangular filter is installed in the 4*f* optical system to reserve first-order diffraction light. The virtual phase SLM is generated on the image plane and recorded on the material as a hogel. In the study, the hogel is recorded as a transmission-type hologram. When a hogel is recorded, the shutter is closed. Then, the SLM changes the uploaded hologram, and the stage moves simultaneously to record the next hogel. The moving distance of the stage is determined by the size of the SLM. Before recording the next hogel, the printing process is halted for a short time for stabilization. The parameters for the holographic material and the printing HOE are described in Table 2 and Table 3.

### 2.4. The Algorithm for Generating Synthetic Hologram

**Figure 4:**
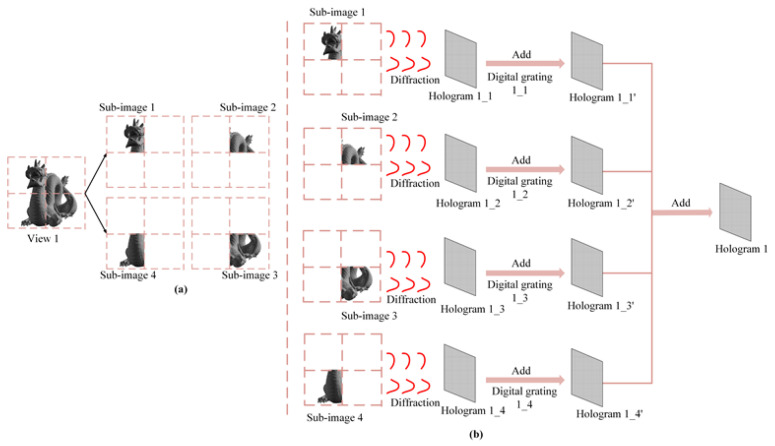

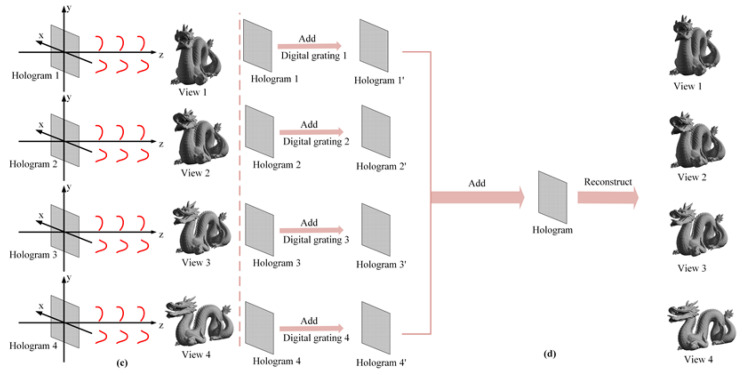
The schematic diagram of the synthetic hologram generation process. (**a**) the separation process of sub-images for view 1 (**b**) the generation process of hologram for view 1 (**c**) the Correspondence between holograms and viewpoints (**d**) the generation process for the synthetic hologram.

The schematic diagram of the synthetic hologram generation process is described in Figure 4. The information sources are four-view images of the 3D scene, which are obtained by the 3D computer graphic rendering technique. Each view of the 3D scene is sliced into multiple 2D parallel layers ($L_{1},L_{2},\ldots L_{n},\ldots L_{N}$) with depth cues along the direction perpendicular to the hologram plane, in which $L_{n}$ denotes the n*th* layer.

The generation process of the hologram is divided into two parts. The first part is achieving the splicing of the reconstruction. In the study, the resolution of the SLM is 1920×1080, and the resolution of each perspective is 3840×2160. Figure 4a denotes the separation process of the viewpoint, taking viewpoint 1 as an example. Viewpoint 1 with a resolution of 4K is divided into four sub-images with a resolution of 2K. According to the angular spectrum diffraction theory, the complex amplitude distribution of each sub-image for each perspective can be calculated by:

(3) $$E_{imn}(x,y)=F^{-1}\left\{F\left\{A_{imn}(x,y)\right\}\exp[j\frac{2\pi }{\lambda }z_{imn}\sqrt{1-{\left(\lambda f_{x}\right)}^{2}-{\left(\lambda f_{y}\right)}^{2})}]\right\}$$

where F represents fast Fourier Transform, $F^{-1}$ represents the inverse Fourier Transform. *λ* is the wavelength of the incident wave. i(i=1,2,…,M) denotes the number of perspectives, *m* denotes the number of the sub-image for each perspective, *n* is the number of sliced layers. $A_{imn}(x,y)$ denotes the complex amplitude distribution of the 3D scene. *z_imn_* is the propagation distance between the n*th* layer plane and the hologram plane, $f_{x}=x/\lambda z_{imn}$ and $f_{y}=y/\lambda z_{imn}$. *j* denotes the imaginary unit. The phase-only hologram is calculated as follows:

(4) $$\varphi_{imn}(x,y,z)=\arg[E_{imn}(x,y)]$$

where *arg* represents the argument of the complex amplitude distribution. Figure 4b represents the generation process of the hologram for viewpoint 1. The generation processes of other viewpoints are similar to Figure 4b.

To achieve seamless splicing of the reconstructed images, digital grating is added in the algorithm. The phase distribution of the digital grating is calculated as follows:

(5) $$\varphi_{DG}=\frac{2\pi }{T}\mathrm{mod}(pr+qs,T)$$

where *mod* represents the modulo operation, *T* denotes the period of the digital grating, *r* and *s* are the vertical and horizontal ranges of the digital grating, respectively. *p* and *q* are the grating loaded onto *r* and *s* directions, and the orientation of the grating can be changed by adjusting the values of *p* and *q*. Hence, the seamless splicing of the reconstructed images can be achieved by setting appropriate values for *p* and *q*. Since the maximum phase modulation of the SLM is 2π, so the phase distribution of the newly obtained hologram after adding the digital grating can be expressed as:

(6) $$\varphi_{new\_imn}=\mathrm{mod}(\varphi_{imn}+\varphi_{DG},2\pi )$$

The phase distribution of the 3D scene for each perspective on the hologram plane can be expressed as:

(7) $$\varphi_{i}=\mathrm{mod}(\sum_{m}^{C\times D}\sum_{n}^{N}\varphi_{new\_imn},2\pi )$$

The second part is adjusting the position of the four perspectives to avoid crosstalk in the reconstruction process, as shown in Figure 4c,d. The digital grating is added in the algorithm analogously, which is referred to as Equations (5) and (6), and the phase distribution for each perspective after adding the digital grating can be calculated by:

(8) $$\varphi_{new\_i}=\mathrm{mod}(\varphi_{i}+\varphi_{DG},2\pi )$$

The phase distribution for the synthetic hologram can be calculated by:

(9) $$\varphi =\mathrm{mod}(\sum_{i}^{M}\varphi_{new\_i},2\pi )$$

**Figure 5:**
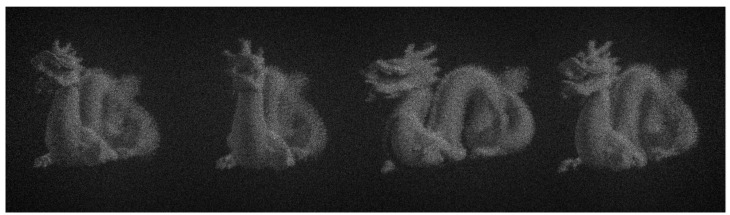
The simulated result of the four perspectives.

The simulated result of the four perspectives is described as shown in Figure 5. Four perspectives are generated simultaneously without crosstalk by setting different values of *p* and *q*.

## 3. Experimental Results

### 3.1. Prototype of the Proposed Multiview Holographic Display

**Figure 6:**
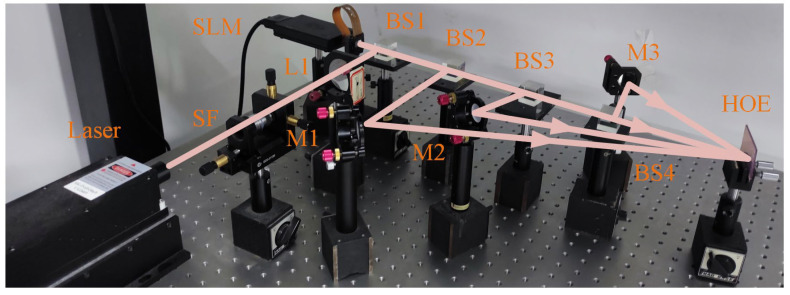
The prototype of the proposed multiview holographic 3D display.

Figure 6 shows the prototype of the proposed multiview holographic 3D display. A red laser (639 nm) is employed as an optical source. The spatial filter (SF), composed of an objective lens and a pinhole, is employed to expand the laser beam. Lens 1(L1) is employed to collimate the laser beam as a plane wave. Multiview synthetic hologram is uploaded on the SLM. Then, the reconstructed wave is split into four waves by beam splitter 2(BS2), beam splitter 3(BS3), and beam splitter 4(BS4). As illustrated in Section 2.1, the direction of the reference wave is different in the recording process of the HOE. The angles of the references wave are set to −20°, −10°, 10°, 20°. Therefore, the incident angle of reconstructed waves should be adjusted to satisfy the reconstruction condition. Here, the incident angle refers to the angle between the probe wave and the normal HOE. The HOE is tilted to adjust the incident angle of the probe wave with 10°. At the same time, M1, M2, and M3 are used to adjust the incident angles of the probe wave with −20°, −10°, and 20°.

The diffraction efficiency, which is measured by the power meter, is an important parameter for the recorded HOE. According to Kogelink’s coupled-wave theory, the diffraction efficiency of the recorded transmission-type HOE is calculated by [24]:

(10) $$\eta =\frac{I-I_{T}}{I}$$

where *I* represents the intensity of the wave incident onto the HOE, *I_T_* represents the intensity of the transmission wave. The absorption and reflection of the medium and the glass substrate are not considered. The diffraction efficiency of the four views is 45.4%. The average diffraction efficiency of each perspective is 11.35%.

### 3.2. Experimental Results with High Resolution

**Figure 7:**
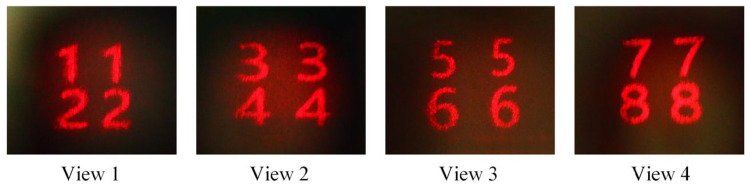
The four-view reconstructions.

In this section, two groups of experiments are performed to verify the effectiveness of the proposed method to realize multiview holographic AR 3D display. In the first experiment, four groups of figures (“1122”, “3344”, “5566”, “7788”) with the resolution of 3840×2160 are employed as four perspectives to project into the recorded HOE. The synthetic phase-only hologram of the four-view is generated according to Section 2.4 and displayed on SLM for optical reconstruction. After modulating the angles of the four perspectives to incident onto the HOE, the four-view reconstructions are captured by moving the digital camera, which is shown in Figure 7. From the captured results, it is clearly seen that there is no crosstalk when observing from the four perspectives. Therefore, the proposed system can realize multiview holographic display with the resolution of 3840×2160 for each perspective.

**Figure 8:**
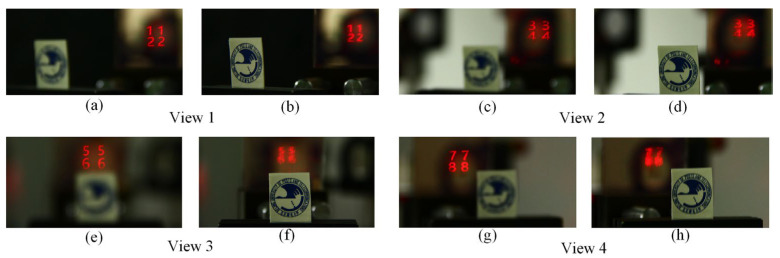
The AR effect of view 1 (**a**) the camera focused on the figures (**b**) the camera focused on the school badge. The AR effect of view 2 (**c**) the camera focused on the figures (**d**) the camera focused on the school badge. The AR effect of view 3 (**e**) the camera focused on the figures (**f**) the camera focused on the school badge. The AR effect of view 4 (**g**) the camera focused on the figures (**h**) the camera focused on the school badge.

Furthermore, to demonstrate the AR effect in the multiview holographic display system, the school badge of the Beijing University of Posts and Telecommunications (BUPT) is used as a real object. From the captured results shown in Figure 8a–h, it is seen that the figures are focused on which school badge is dim, as shown in Figure 8a,c,e,g. In the same situation, the school badge becomes clear in which the figures are defocused, as shown in Figure 8b,d,f,h. Thus, the experimental results certify that the proposed system can implement see-through multiview holographic AR display that mixes virtual images with the real scene.

**Figure 9:**
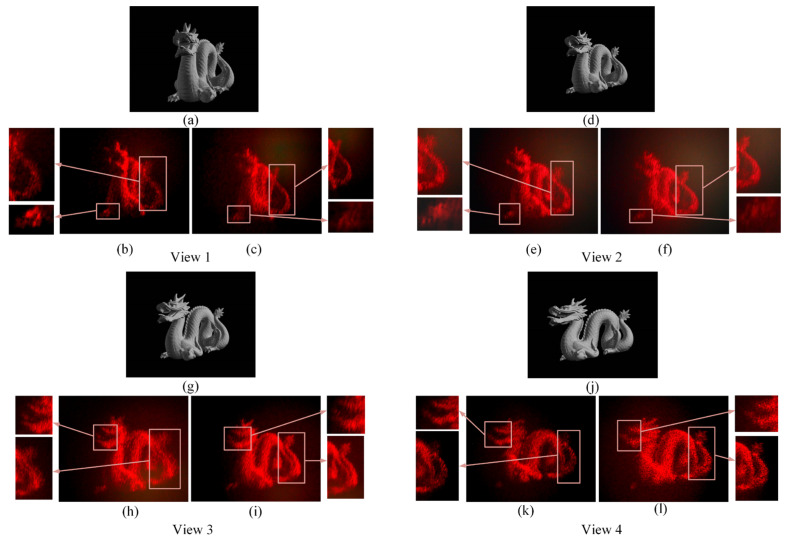
(**a**) The image of view 1. The captured reconstructed image of view 1 (**b**) the camera focused on the claw (**c**) the camera focused on the tail (**d**) The image of view 2. The captured reconstructed image of view 2 (**e**) the camera focused on the claw (**f**) the camera focused on the tail (**g**) The image of view 3. The captured reconstructed image of view 3 (**h**) the camera focused on the mouth (**i**) the camera focused on the tail (**j**) The image of view 4. The captured reconstructed image of view 4 (**k**) the camera focused on the mouth (**l**) the camera focused on the tail.

To further verify the effectiveness of the proposed method to realize multiview holographic AR display with high resolution for each perspective, we designed a complicated 3D scene composed of a dragon model and obtain the four-view images. The synthetic phase-only hologram with a maximum depth of 150 mm was calculated using the proposed method, as described in Section 2.4, and then uploaded onto SLM for holographic reconstruction. The amplitude information for view 1 is shown in Figure 9a. Figure 9b,c shows the captured reconstructed images at different focus depths for view 1, where the former and the latter are focused on the claw and the tail of the dragon, respectively.

Analogously, Figure 9d shows the amplitude information for view 2. Figure 9e,f shows the captured reconstructed images at different focus depths for view 2, where the former and the latter are focused on the claw and the tail of the dragon, respectively. In the same way, Figure 9g shows the amplitude information for view 3. Figure 9h,i shows the captured reconstructed images at different focus depths for view 3, where the former and the latter are focused on the mouth and the tail of the dragon, respectively. Figure 9j shows the amplitude information for view 4. Figure 9k,l shows the captured reconstructed images at different focus depths for view 4, where the former and the latter are focused on the mouth and the tail of the dragon, respectively. Thus, it can also be seen that the four-view reconstructed 3D images have real physical depth such that the observer can effectively perceive the true 3D sensation without accommodation-verge conflict. No crosstalk can be observed in the system. The above experimental results demonstrate that the proposed system can achieve multiview holographic display successfully without the visual fatigue problem. The resolution for each view is 3840×2160. Hence, the proposed method can realize multiview holographic display with high resolution.

**Figure 10:**
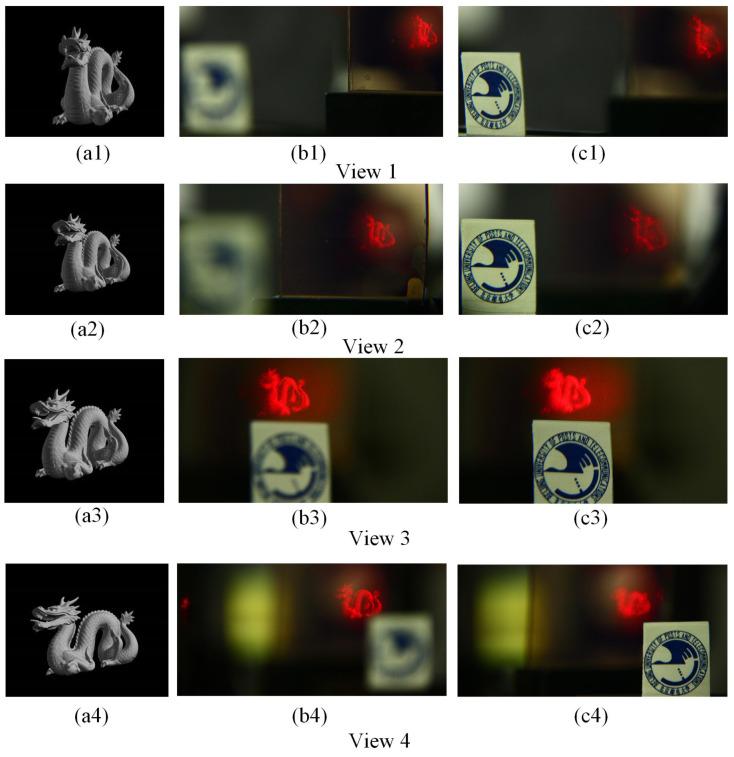
(**a1**) The image of view 1. The captured reconstructed image of view 1 (**b1**) the camera focused on the tail (**c1**) the camera focused on the emblem of the BUPT (**a2**) The image of view 2. The captured reconstructed image of view 2 (**b2**) the camera focused on the tail (**c2**) the camera focused on the emblem of the BUPT (**a3**) The image of view 3. The captured reconstructed image of view 3 (**b3**) the camera focused on the tail (**c3**) the camera focused on the emblem of the BUPT (**a4**) The image of view 4. The captured reconstructed image of view 4 (**b4**) the camera focused on the tail (**c4**) the camera focused on the emblem of the BUPT.

Furthermore, in order to better demonstrate the multiview holographic AR 3D display with the proposed system, the emblem of BUPT as a real object is placed in the optical path to express the real physical depth and parallax effect. Figure 10(b1,c1) shows the captured reconstructed image for view 1 at different focus depths, where the former and the latter are focused on the tail of the dragon and the emblem of the BUPT, respectively. Analogously, Figure 10(b2,c2) shows the captured reconstructed image for view 2, where the former and the latter are focused on the tail of the dragon and the emblem of the BUPT, respectively. Figure 10(b3,c3) shows the captured reconstructed image for view 3, where the former and the latter are focused on the tail of the dragon and the emblem of the BUPT, respectively. Figure 10(b4,c4) shows the captured reconstructed image for view 4, where the former and the latter are focused on the tail of the dragon and the emblem of the BUPT, respectively. From the captured results Figure 10(b1,c1), or Figure 10(b2,c2), or Figure 10(b3,c3), or Figure 10(b4,c4), we can confirm that the proposed system can realize multiview holographic AR 3D display successfully. Besides, from the captured results in Figure 10(b1–b4), or Figure 10(c1–c4), the relative position between the dragon and the emblem of the BUPT is different, which demonstrates that the proposed system can achieve a horizontal parallax effect. Hence, the proposed system can achieve both holographic AR effect and horizontal parallax at the same time.

**Figure 11:**
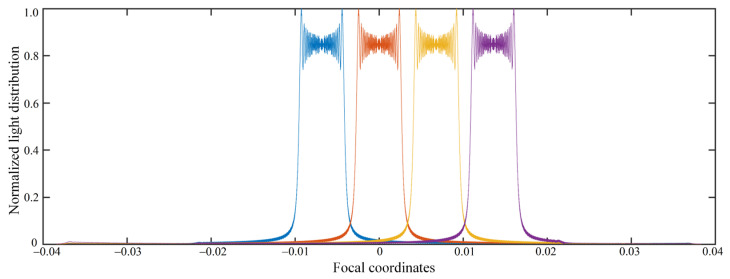
Normalized light distribution for the four perspectives.

Besides, we conducted a study on the crosstalk between the four perspectives. When the viewing distance was 75 cm, we simulated the normalized light distribution for four perspectives, as shown in Figure 11. Four different color lines represent the light distribution for four perspectives, respectively. It can be seen there are no overlapping areas of light distribution. Hence, there is no crosstalk between the four perspectives.

**Figure 12:**
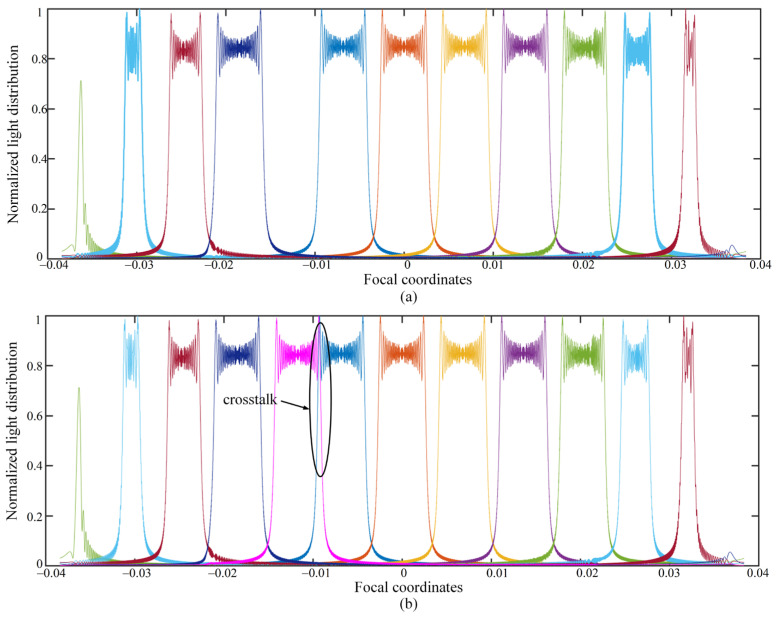
Normalized light distribution (**a**) for the eight perspectives, (**b**) for the nine perspectives.

Limited by the size of HOE, the method we proposed can only achieve eight perspectives holographic display at most, as shown in Figure 12a. For nine perspectives holographic display, the light distribution overlaps, which reveals that the crosstalk would generate, as shown in Figure 12b.

## 4. Conclusions and Discussion

In summary, we propose a method to realize multiview holographic AR 3D display based on the HOE. The core component is HOE which is employed to redirect the multiview reconstructed images into multiple viewing zones. The SLM is employed to upload the synthetic phase-only hologram generated by the angular spectrum diffraction theory. Digital grating is introduced in the generation process of the hologram to achieve the splicing of the reconstructions and adjust the positions of the reconstructions. The experiment results verify that our proposed system can achieve multiview holographic AR 3D display without crosstalk. The resolution for each viewpoint is 4K, which is higher than that of the existing multiview holographic display system.

For future work, more beam splitters will be employed to achieve multiview holographic display with more viewpoints. Due to the attenuation characteristics of beam splitters, the brightness of the reconstructed image decreases with more viewpoints. To solve the problem, we should study how to increase the diffraction efficiency of the HOE to improve the display performance of the holographic display system. The HOE is expected to be used in window displays, exhibitions, and education in the future.

## Figures and Tables

**Table 1:** The detailed design parameters of the HOE.

Parameter	Values
Focal point 1	(−5, 10, 550 mm)
Focal point 2	(0, 10, 550 mm)
Focal point 3	(5, 10, 550 mm)
Focal point 4	(10, 10, 550 mm)
Focal length	550 mm
Viewing range	(−20°, 20°)

**Table 2:** The parameters of the printing HOE.

Parameter	Values
Sensitive wavelength	633, 532, 457 nm
Photosensitivity	150 mJ/cm^2^ @633 nm
Refractive index modulation	>0.02
Refractive index of material	1.47
Photosensitive layer thickness	18 ± 1 um

**Table 3:** The parameters of the printing HOE.

Parameter	Values
Pixel pitch of SLM	8 um
Resolution of SLM	1920 × 1080
Resolution of CGH	3840 × 2160
Resolution of hogel	1920 × 1080
Number of hogels	2 × 2
Exposure time/hogel	73.7 s
Exposure energy/hogel	150 mJ
Size of a hogel Size of HOE	15.36 × 8.64 mm 30.72 × 17.28 mm
Total exposure time	294.8 s

## Data Availability

Data is contained within the article.
